# Local Effect of Quinine in Diphtheria

**Published:** 1878-01

**Authors:** 


LOCAL EFFECT OF QUININE IN DIPHTHERIA.—Dr. E. J. Zinke, of Cincinnati, in the *Clinic*, extols the use of quinia as a topical application in diphtheria. His experience is confined to three cases, but the success of the remedy was the more marked from the fact that these three recovered while four cases treated in the orthodox manner immediately before, terminated fatally. The manner of using it was in solution and applied by means of an atomizer; strength, a drachm of the salt to an ounce of water, with enough hydrochloric acid to make a clear solution. Quinia acts by killing the micrococci, masses of which, betraying no signs of life, were discovered by Dr. Zinke in membranes thrown off in the cases reported.

